# Catalytic Tunnel Dynamics Explain Functional Divergence of FAD2 Homologs Underlying the Oleic-to-Linoleic Ratio in *Cornus wilsoniana*

**DOI:** 10.3390/biology15020145

**Published:** 2026-01-14

**Authors:** Wenyan Yuan, Wei Wu, Shushan Jia, Yunzhu Chen, Peiwang Li, Yan Yang, Lijuan Jiang, Qiang Liu, Li Li, Wenbin Zeng, Xiao Zhou, Jingzhen Chen

**Affiliations:** 1 State Key Laboratory of Woody Oil Resources Utilization, Hunan Academy of Forestry, Changsha 410004, China; 20231100133@csuft.edu.cn (W.Y.); 20221200173@csuft.edu.cn (W.W.); cyzcarol@163.com (Y.C.);; 2 College of Life Science and Technology, Central South University of Forestry and Technology, 498 South Shaoshan Road, Changsha 410004, China; 3 Qingdao Research Academy of Environmental Sciences, Qingdao 266000, China; w2062055326@163.com

**Keywords:** *Cornus wilsoniana*, FAD2, molecular dynamics simulation, catalytic tunnel

## Abstract

The oleic-to-linoleic acid ratio (O/L) is a key determinant of oil quality, yet its molecular basis in *Cornus wilsoniana* remains unclear. Here, we combined fatty-acid profiling with molecular dynamics (MD) simulations and catalytic tunnel analysis to compare four annotated FAD2 homologs. Sequence alignment revealed a major variable segment at residues 160–185, including a small deletion in CW09G04700 and an extensive deletion in CW04G07690. Docking against oleic acid supported excluding CW04G07690 due to weak binding. Eighty-nanosecond MD simulations showed that CW02G01750 and CW09G27260 rapidly converged to stable conformational ensembles with lower core flexibility, whereas CW09G04700 exhibited higher internal mobility around residues 180–220. CAVER analysis further indicated increasingly accessible catalytic tunnels for CW02G01750 and CW09G27260 during simulation, while CW09G04700 displayed transient tunnel narrowing accompanied by ligand conformational readjustments. These results nominate CW02G01750 as a leading structural candidate among *C. wilsoniana* FAD2 homologs and highlight access-pathway dynamics as a mechanistic feature potentially contributing to O/L formation.

## Simple Summary

High-quality edible oils usually contain more oleic acid and less linoleic acid, but the molecular reasons for this balance remain unclear in the woody oil tree *Cornus wilsoniana*. We measured the main fatty acids in its fruit oil and then compared four closely related versions of an enzyme called fatty acid desaturase 2, which converts oleic acid into linoleic acid. One version lacked a large protein segment and showed a damaged binding pocket, suggesting it is unlikely to function properly. For the remaining three versions, we used molecular dynamics simulations together with catalytic tunnel tracking, a way to follow the passageway that guides the fatty acid to the reactive center, to watch how the protein shape changes during binding. Two versions formed a stable, more open access tunnel that kept the substrate well positioned, whereas the third showed higher internal flexibility and a tunnel that narrowed later in the simulation, forcing repeated substrate readjustments. These results prioritize CW02G01750 as the leading structural candidate among annotated *C. wilsoniana* FAD2 homologs and suggest that access-pathway dynamics may modulate substrate accommodation, providing a mechanistic hypothesis for future validation of O/L regulation.

## 1. Introduction

*Cornus wilsoniana* is an emerging woody oil crop with substantial ecological and economic value [1]. The fruit oil is characterized by a high proportion of C18 unsaturated fatty acids, particularly oleic acid (C18:1) and linoleic acid (C18:2), which together largely determine nutritional quality, oxidative stability, and the storage performance of plant oils [2,3,4]. Among these traits, the oleic-to-linoleic acid ratio (O/L ratio) is widely recognized as a pivotal indicator linking lipid composition to both health-related attributes and technological properties [5,6]. Therefore, elucidating the molecular basis governing the O/L ratio in *C. wilsoniana* is not only important for understanding lipid metabolism in woody oil species but also valuable for breeding and targeted improvement of oil quality.

Fatty acid desaturation constitutes the key biochemical route shaping the C18 fatty-acid profile [7]. In higher plants, the conversion of oleic acid to linoleic acid is predominantly catalyzed by microsomal ω-6 fatty acid desaturase (FAD2), which operates as a rate-limiting step that directly sets the O/L ratio [8,9,10]. In our previous chromosome-level genome study of *C. wilsoniana*, we systematically annotated genes related to fatty acid desaturation and identified four candidate FAD2 homologs (CW02G01750, CW09G27260, CW09G04700, and CW04G07690), providing a solid genomic foundation for functional dissection [11]. However, the relative contribution of these FAD2 homologs to oil formation—particularly during the rapid oil-accumulation stage—remains unclear, and the mechanistic basis underlying potential functional divergence among the four FAD2 proteins has not yet been resolved.

In addition to enzyme identity, the O/L ratio can also be influenced by the expression level and developmental timing of individual FAD2 homologs, substrate availability in cellular acyl-lipid pools, and the endoplasmic-reticulum membrane environment that constrains substrate access and tunnel geometry. Although these factors were outside the scope of the present work, we acknowledge their importance when interpreting simulation-based prioritization of candidate FAD2s.

Although multiple enzymes participate in the C18 desaturation network, not all steps contribute equally to variation in the O/L ratio of *C. wilsoniana* oil [12]. In contrast to the direct role of FAD2 in controlling the flux from C18:1 to C18:2, upstream and downstream steps are less informative for explaining O/L differences when their corresponding fatty-acid pools remain relatively low or stable in mature oil [13,14]. Consequently, focusing on FAD2 represents the most parsimonious and mechanistically grounded strategy to interpret and regulate the O/L ratio in *C. wilsoniana*, while simultaneously enabling a clear linkage from gene identity to biochemical outcome.

Accordingly, this study aims to prioritize candidate FAD2 homologs that may contribute to the oleate-to-linoleate conversion step relevant to the O/L ratio in *C. wilsoniana* oil by using a comparative structural and dynamic workflow. First, we quantified the fatty-acid composition of *C. wilsoniana* oil by gas chromatography, thereby defining the relative abundance of oleic and linoleic acids and the characteristic O/L ratio. Building on this phenotypic profile, we performed homology modeling and multiple sequence alignment of the four candidate FAD2 proteins to pinpoint divergent segments and putative key residues. We then conducted molecular docking to compare substrate-binding affinities among FAD2 homologs and examined differences in the geometry of the catalytic pocket to structurally assess substrate recognition and positioning. Finally, molecular dynamics simulations were employed to characterize the dynamic binding behavior and stability of the protein-substrate complexes, with additional analyses focusing on potential catalytic substrate-access tunnels. By integrating fatty-acid phenotype, sequence divergence, three-dimensional structure, and substrate binding dynamics, our work links FAD2 sequence variation with substrate accommodation and access-pathway behavior, thereby providing a structure-informed basis for prioritizing targets for future functional validation and oil-quality improvement.

## 2. Materials and Methods

### 2.1. Materials

Fruits of *Cornus wilsoniana* were collected from the National Tree Breeding Base at the Experimental Forest Farm of the Hunan Academy of Forestry (Changsha, China). The harvested samples were rapidly frozen in liquid nitrogen and stored at −80 °C prior to subsequent analyses.

Fatty-acid standards were obtained from Sigma-Aldrich (St. Louis, MO, USA). Solvents of chromatographic grade and other chemicals of analytical grade were used for fatty-acid methyl ester (FAME) preparation and GC analysis. Fatty-acid profiling was conducted using a Nexis GC-2030 gas chromatograph equipped with a flame ionization detector (Shimadzu, Kyoto, Japan); other routine laboratory instruments were used as needed.

### 2.2. Extraction and Methylation of Fatty Acids

Fatty acid methyl esters (FAMEs) were prepared from *C. wilsoniana* fruit oil using an alkaline methylation approach. Briefly, 0.06 g of oil was dissolved in 4.0 mL isooctane in a 10 mL centrifuge tube, followed by the addition of 2.0 mL KOH–methanol solution (2 mol/L). The mixture was vigorously shaken for 60 s to allow methylation.

To terminate the reaction and facilitate phase separation, 1.0 g anhydrous NaHSO_4_ was added, and the tube was shaken for another 30 s and left standing for 10 min. The upper organic layer was collected, passed through a 0.22 μm organic membrane, and used for GC analysis.

### 2.3. Gas Chromatography Analysis (GC–FID)

FAMEs were analyzed on a Nexis GC-2030 gas chromatograph (Shimadzu Corporation, Kyoto, Japan) equipped with a flame ionization detector and a fused-silica capillary column (0.25 mm × 100 m, 0.25 μm film thickness). Nitrogen served as the carrier gas at 1.1 mL/min, and samples were introduced in split mode (split ratio 1:100). The injector temperature was maintained at 270 °C, and 1.0 μL was injected for each run. GC–FID analyses were conducted using three independent biological replicates, each prepared from fruits collected from different individual trees.

The oven program was as follows: 100 °C for 13 min; ramp to 180 °C at 10 °C/min and hold for 6 min; ramp to 200 °C at 1 °C/min and hold for 20 min; then ramp to 230 °C at 4 °C/min and hold for 10.5 min. Fatty acids were identified by matching retention times against the Supelco 37 Component FAME Mix (Supelco, Bellefonte, PA, USA). Relative fatty-acid contents were calculated by normalizing individual peak areas to the total integrated peak area.

### 2.4. Homology Modeling and Multiple Sequence Alignment

Homology models of the four candidate FAD2 proteins were generated using the SWISS-MODEL server (https://swissmodel.expasy.org/) accessed on 25 November 2025. Model quality was primarily evaluated using the Global Model Quality Estimation (GMQE) score provided by SWISS-MODEL, and stereochemical reasonability was further assessed using Ramachandran plots generated via the SAVES v6.1 server (https://saves.mbi.ucla.edu/) accessed on 25 November 2025. Multiple sequence alignment of the four FAD2 proteins was performed, and the alignment was visualized using ESPript 3.2 (https://espript.ibcp.fr/ESPript/ESPript/) accessed on 26 November 2025 with emphasis on the divergent regions among the four sequences.

For homology modeling, SWISS-MODEL selected AlphaFold DB templates as follows: CW02G01750 and CW09G27260 were modeled using template I1N982.1.A (sequence identity 81.98% and 80.16%, respectively; coverage 100%); CW09G04700 used template Q8GZC3.1.A (sequence identity 79.89%; coverage 100%); CW04G07690 used template A0A5J4ZHK7.1.A (sequence identity 80.31%; coverage 99.7%).

### 2.5. Molecular Docking

Molecular docking was performed to compare the binding affinity of the four modeled FAD2 proteins toward the representative ligand (oleic acid, C18:1) and to characterize the catalytic pocket involved in substrate binding. The 3D structure of the ligand was obtained from the PubChem database and prepared prior to docking.

Docking calculations were carried out using AutoDock Vina (v1.1.2) [15]. Receptor and ligand structures were processed with AutoDockTools (e.g., hydrogen addition, Gasteiger charge assignment, and conversion to PDBQT format). A cubic grid box (50 × 50 × 50 Å) was applied to cover the catalytic region, and the best-scoring pose (lowest binding energy) was retained for subsequent pocket comparison and structural interpretation. Docking poses were inspected and visualized using VMD (v1.9.2). Protein–ligand interaction diagrams were generated using LigPlot+ (v2.2.9) to summarize key contacts within the catalytic pocket [16].

The docking search space was defined by cavity prediction on each modeled FAD2 structure. The grid box was centered on the centroid of the predicted substrate-binding cavity and sized to fully encompass the catalytic pocket and accommodate oleic acid.

### 2.6. Molecular Dynamics Simulation and Catalytic Tunnel Prediction

Molecular dynamics (MD) simulations were carried out with GROMACS 2020.6 [17]. The CHARMM36 force field was used for the FAD2 proteins, and ligand topology/parameters were generated using the CGenFF 3.0.1 webserver (https://cgenff.com) accessed on 7 December 2025. CGenFF penalty scores were inspected to assess parameter transferability; all penalties were below 10, with a maximum penalty score of 3.083, indicating a fair analogy for oleic acid. The CGenFF stream file is provided in the Supplementary Materials. Each FAD2–ligand complex was solvated in an orthorhombic box with TIP3P water, followed by the addition of Na^+^/Cl^−^ ions to neutralize the system. Energy minimization was performed using the steepest-descent algorithm (5000 steps), and the system was subsequently equilibrated under NVT and NPT ensembles (100 ps each) at 300 K and 1 atm. Production simulations were run for 80 ns with a 2 fs time step. For each complex, one 80 ns trajectory was generated; therefore, the MD-based comparisons are intended to be qualitative and trend-focused. Convergence was assessed by monitoring the stabilization of RMSD, Rg, and SASA profiles. The LINCS algorithm was applied to constrain bond lengths, and long-range electrostatics were treated using the particle-mesh Ewald (PME) method.

Trajectory convergence was evaluated by monitoring three global descriptors: backbone RMSD (nm), radius of gyration (Rg, nm), and solvent-accessible surface area (SASA, nm^2^). A quasi-equilibrated regime was defined as a continuous time window showing no systematic drift in these descriptors and only fluctuations around stable mean values.

Catalytic tunnel prediction was performed using the CAVER 3.0.3 plugin, enabling comparative analysis of putative substrate-access channels among different FAD2 variants. To improve transparency and reproducibility, identical CAVER settings were applied to all snapshots: minimum probe radius 0.9 Å, shell depth 4 Å, shell radius 3 Å, clustering threshold 3.5 Å, and 12 approximating balls. The starting point was defined as the centroid of a predefined atom selection near the catalytic pocket center; for each snapshot, the centroid coordinates were recalculated using the same selection criteria. Starting-point optimization used a maximum distance of 3 Å and a desired radius of 5 Å. Tunnels were ranked by throughput, and the dominant tunnel was defined as the path with the highest throughput in each snapshot.

## 3. Results

### 3.1. Fatty-Acid Composition of Cornus Wilsoniana Oil

**Figure 1:**
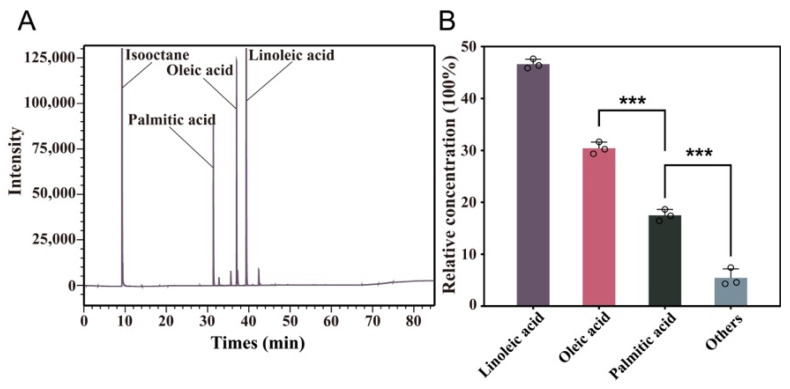
GC-FID profiling of major fatty acids in *Cornus wilsoniana* oil. (**A**) Representative GC-FID chromatogram of fatty acid methyl esters (FAMEs) from *C. wilsoniana* oil, with major peaks annotated (isooctane, linoleic acid, oleic acid, and palmitic acid). (**B**) Relative abundance of the major fatty-acid components in *C. wilsoniana* oil. Data are presented as mean ± SD of biological replicates (n = 3). Asterisks indicate significant differences (***, *p* < 0.001).

Gas chromatography profiling revealed a simple fatty-acid pattern in *Cornus wilsoniana* oil, with several dominant peaks corresponding to major components (Figure 1A). The chromatogram showed clearly resolved signals for linoleic acid, oleic acid, and palmitic acid, with isooctane appearing as the solvent peak.

Quantitative analysis indicated that linoleic acid was the most abundant fatty acid (46.03%), followed by oleic acid (30.25%) and palmitic acid (17.43%), whereas the remaining fatty acids collectively accounted for less than 7% of the total (Figure 1B). Notably, linoleic acid and oleic acid were present at significantly higher levels than palmitic acid and the minor components, highlighting a C18 unsaturated fatty-acid-enriched profile in *C. wilsoniana* oil.

### 3.2. Sequence Divergence and Structural Differences Among Four FAD2 Homologs

**Figure 2:**
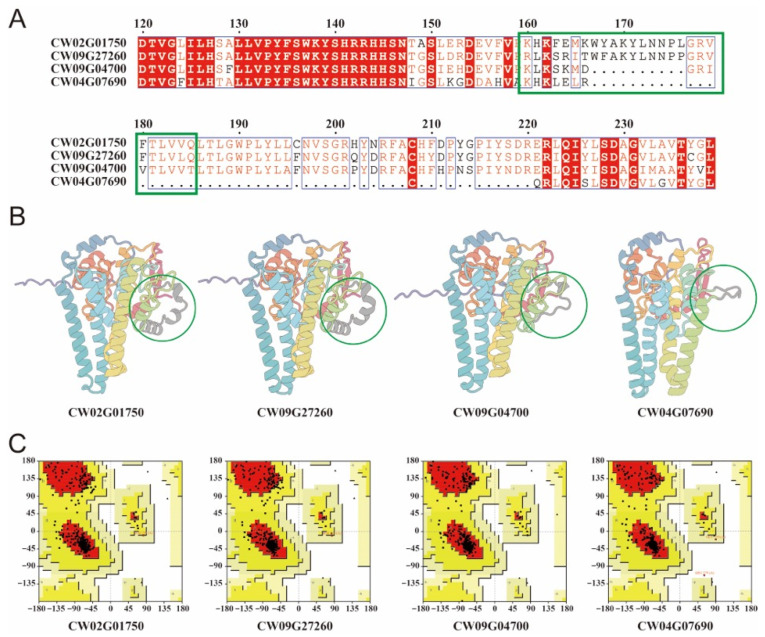
Sequence alignment and homology modeling of four *C. wilsoniana* FAD2 proteins. (**A**) Multiple sequence alignment of four FAD2 homologs; the major divergent segment is highlighted in green. (**B**) Homology models of the four FAD2 proteins, with the divergent region indicated by green circles. (**C**) Ramachandran plot validation of the modeled structures, showing the distribution of residues in allowed regions.

Multiple sequence alignment of the four *C. wilsoniana* FAD2 homologs revealed that sequence divergence was mainly confined to a localized segment, with the most prominent variations occurring within the region spanning approximately residues 160–185 (Figure 2A). Within this divergent segment, CW09G04700 exhibited a small deletion, whereas CW04G07690 contained an extensive deletion, suggesting that the integrity of this region may differ substantially among the four homologs and potentially contribute to functional variation. The amino-acid sequences of the four FAD2 proteins are provided in the Supplementary Materials.

Consistent with the alignment results, homology models showed that CW02G01750 and CW09G27260 adopted highly similar overall conformations, while structural deviations were primarily observed at the divergent region highlighted in Figure 2B. In CW09G04700, the corresponding α-helical element in this region appeared to transition into a more flexible coil, whereas CW04G07690 displayed partial loss of secondary-structure elements, in agreement with the larger deletion detected by sequence alignment. Model quality assessment using Ramachandran plots indicated that all four models had more than 90% of residues located in favored or allowed regions (Figure 2C), supporting the reliability of the modeled structures for subsequent comparative analyses [18].

### 3.3. Molecular Docking Reveals Differential Binding of Oleic Acid to Four FAD2 Homologs

**Figure 3:**
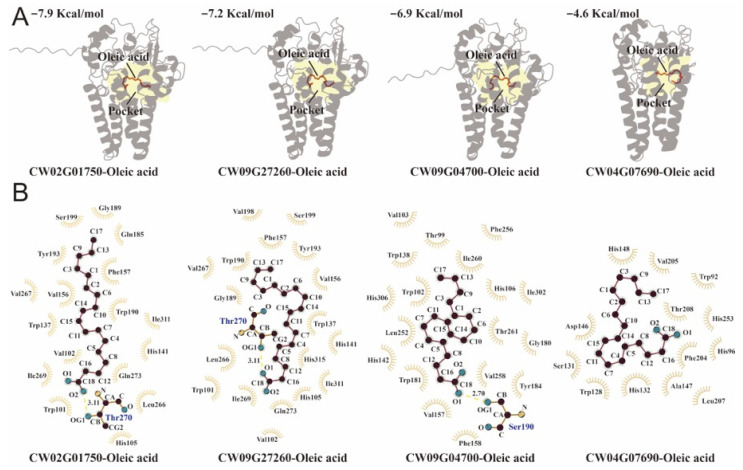
Docking of oleic acid to four *C. wilsoniana* FAD2 proteins. (**A**) Representative docking poses of oleic acid in the catalytic pocket of each FAD2 model; the pocket region is highlighted in yellow and docking binding energies are indicated. (**B**) Two-dimensional protein–ligand interaction diagrams showing key contacts between oleic acid and the catalytic pocket residues.

Docking analysis showed that oleic acid could be accommodated within the catalytic pocket of each modeled FAD2 protein (Figure 3A). The predicted binding affinities were −7.9 kcal/mol for CW02G01750, −7.2 kcal/mol for CW09G27260, −6.9 kcal/mol for CW09G04700, and −4.6 kcal/mol for CW04G07690. These results indicate the strongest interaction for CW02G01750 and the weakest for CW04G07690. In CW04G07690, the pocket region appeared markedly reduced in volume, consistent with its extensive deletion in the divergent segment, suggesting that the catalytic environment required for stable substrate binding may be compromised. Here, −5 kcal/mol was treated solely as a practical, AutoDock Vina scoring-specific reference to flag low-ranking poses within our dataset; it is not a universal biophysical threshold for binding, and its interpretation is protein- and protocol-dependent [19]. Accordingly, together with the markedly reduced pocket volume and the absence of stabilizing hydrogen bonds, CW04G07690 was deprioritized and not carried forward to downstream MD analyses.

The 2D interaction maps further supported these differences (Figure 3B). In both CW02G01750 and CW09G27260, Thr270 formed a hydrogen bond with oleic acid with a bond length of 3.11 Å. In CW09G04700, Ser190 formed a hydrogen bond with oleic acid with a bond length of 2.70 Å. In contrast, no hydrogen bond was detected between CW04G07690 and oleic acid, consistent with its weak docking affinity.

### 3.4. Molecular Dynamics Simulations Reveal Distinct Stability and Flexibility Among FAD2–Oleic Acid Complexes

**Figure 4:**
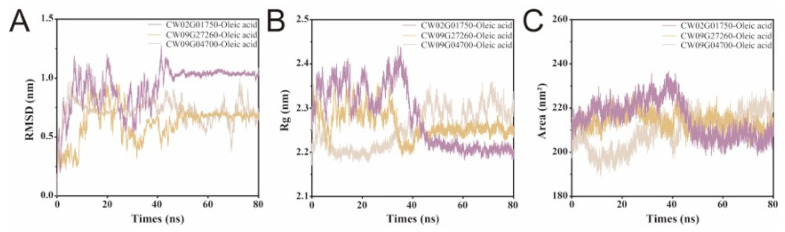
MD trajectory analyses of three FAD2–oleic acid complexes. (**A**) Root-mean-square deviation (RMSD) of the protein backbone during 80 ns simulations. (**B**) Radius of gyration (Rg) over time. (**C**) Solvent-accessible surface area (SASA) over time.

Molecular dynamics simulations were conducted to evaluate the conformational stability and dynamic behavior of the three FAD2–oleic acid complexes retained after docking. Because one trajectory was analyzed for each complex, the results below are reported as trajectory-level comparisons that emphasize consistent trends across multiple observables. Time-dependent RMSD trajectories showed that CW02G01750 and CW09G27260 approached a relatively stable regime at around 50 ns, with CW09G27260 maintaining a lower post-equilibration RMSD than CW02G01750, indicating smaller overall structural deviation during the late stage of the simulation (Figure 4A). In contrast, CW09G04700 exhibited persistent mild fluctuations beyond 50 ns, suggesting increased conformational flexibility [20]. In the quasi-equilibrated segment (50–80 ns), CW02G01750 and CW09G27260 fluctuated within approximately 1.0–1.1 nm and 0.6–0.7 nm RMSD, respectively, whereas CW09G04700 remained more variable at about 0.6–0.9 nm. Consistently, Rg stabilized at approximately 2.20–2.23 nm for CW02G01750, 2.23–2.26 nm for CW09G27260, and 2.27–2.33 nm for CW09G04700, with corresponding SASA plateaus of approximately 205–212, 212–220, and 215–230 nm^2^.

**Figure 5:**
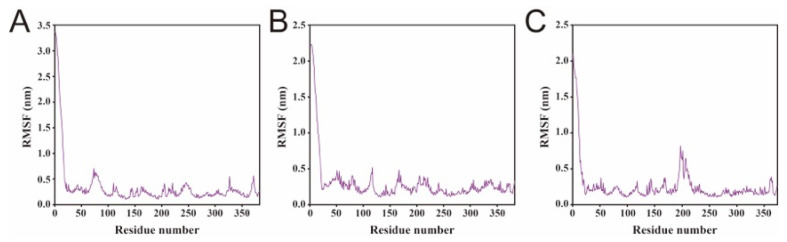
Residue-level flexibility profiles of FAD2–oleic acid complexes from MD simulations. RMSF profiles of (**A**) CW02G01750, (**B**) CW09G27260, and (**C**) CW09G04700 during 80 ns simulations, showing residue-wise fluctuations of the protein backbone in the presence of oleic acid.

Consistent with RMSD, the radius of gyration and solvent-accessible surface area converged for all three complexes at approximately 45 ns, while clear differences remained in their equilibrated levels (Figure 4B,C). CW02G01750 displayed a lower Rg than CW09G27260 after equilibration, indicating a comparatively more compact conformation, whereas CW09G04700 showed the highest Rg and continued to fluctuate slightly in the later phase, accompanied by a similar trend in surface area [21,22]. Residue-level RMSF profiles further highlighted distinct flexibility patterns among the three proteins (Figure 5) [23,24]. CW02G01750 and CW09G27260 exhibited generally low fluctuations across most residues, with higher mobility mainly confined to the N-terminal region. In contrast, CW09G04700 showed elevated RMSF in an internal segment centered around residues 180–220, consistent with the major divergent region identified by sequence/structure comparison. Notably, increased local flexibility within the protein core is more likely to affect functional architecture than terminal motions, as it may perturb the geometry and stability of the catalytic pocket and its surrounding access pathway [25,26,27]. Together, these results indicate that CW09G04700 samples a more dynamic conformational ensemble, whereas CW02G01750 and CW09G27260 maintain comparatively higher structural stability during the simulation.

### 3.5. Conformational Transitions and Catalytic Tunnel Dynamics During MD Simulations

**Figure 6:**
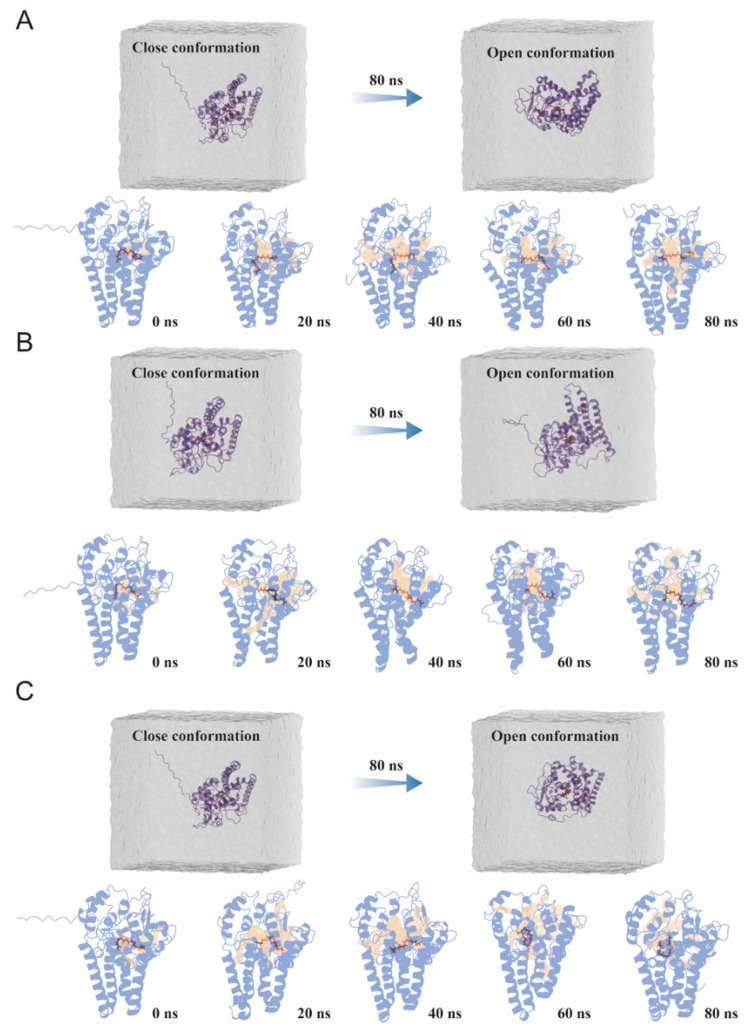
Time-resolved conformational changes and catalytic tunnel dynamics of FAD2–oleic acid complexes during MD simulations. (**A**–**C**) Representative snapshots of CW02G01750–oleic acid, CW09G27260–oleic acid, and CW09G04700–oleic acid complexes along 80 ns MD trajectories. The upper panels show system-level views in the solvent box, illustrating the transition from a relatively closed to an open conformation. The lower panels show complex snapshots extracted at 0, 20, 40, 60, and 80 ns, with predicted catalytic tunnels highlighted in yellow.

To visualize how substrate binding is coupled to structural rearrangements, we tracked the conformational evolution of the three FAD2–oleic acid complexes and the corresponding catalytic tunnels predicted along the MD trajectories (Figure 6). Based on the 80 ns end-point structures, the divergent segment spanning residues 160–185, highlighted in red in Figure S1, is positioned at the rim of the catalytic pocket and adjacent to the entrance of the dominant access tunnel, providing a residue-level structural context for the access-pathway behaviors observed during the trajectories. At the system level, the water-box views indicate a clear transition from a relatively closed state to a more open conformation over the 80 ns simulation, accompanied by increased exposure of the catalytic pocket and stabilization of oleic acid within the active-site region.

**Figure 7:**
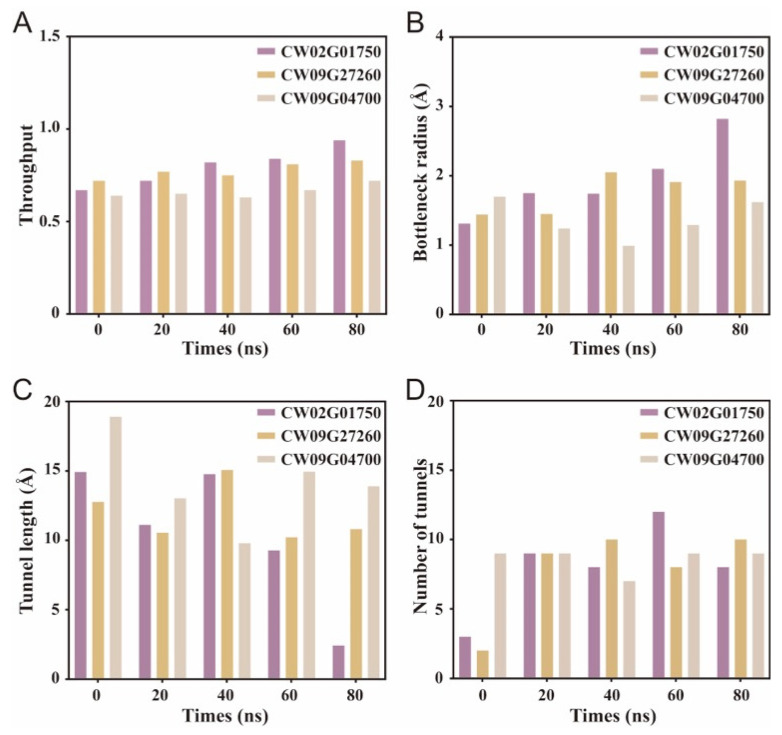
Snapshot-based quantification of catalytic tunnel descriptors during the MD simulations. (**A**) Tunnel throughput score, (**B**) Bottleneck radius, (**C**) Tunnel length, (**D**) Number of detected tunnels. Values were extracted from CAVER outputs at 0, 20, 40, 60, and 80 ns for the CW02G01750, CW09G27260, and CW09G04700 complexes.

Snapshots extracted every 20 ns further highlight distinct dynamic patterns among the three homologs. For CW02G01750 and CW09G27260, oleic acid gradually adopted a more extended conformation and became progressively accommodated in the catalytic pocket as the simulation proceeded. In parallel, the predicted tunnel network expanded and remained more pronounced toward the later stage of the trajectories, consistent with a structural shift toward an open state that facilitates ligand access and positioning. In contrast, CW09G04700 displayed a less consistent binding trajectory, in which oleic acid underwent a sequence of conformational rearrangements from a folded state to a more extended state and then returned to a more compact conformation at the end of the simulation. This behavior coincided with a tunnel pattern that increased during the middle stage but decreased in the late stage, suggesting that transient narrowing of the access pathway may impose additional constraints on ligand accommodation and contribute to a less stable binding mode [28]. These qualitative patterns are further supported by the snapshot-based tunnel descriptors summarized in Figure 7.

To enable objective comparisons, we extracted the CAVER output for each sampled structure and summarized the dominant-tunnel throughput, bottleneck radius, and tunnel length, together with the total number of detected tunnels (Figure 7A–D). Across the sampled snapshots, CW02G01750 exhibited an overall increase in throughput and a marked widening of the bottleneck radius toward the late stage, whereas CW09G27260 remained comparatively high and stable. In contrast, CW09G04700 showed lower throughput with a narrower bottleneck at mid-to-late snapshots, despite a relatively high number of detected tunnels, indicating a less permissive access architecture for sustained ligand accommodation. The dominant tunnel was defined as the highest-throughput tunnel identified by CAVER in each snapshot.

## 4. Discussion

This study provides a structure-informed framework to prioritize candidate FAD2 enzymes potentially underlying the oleic-to-linoleic conversion step associated with the O/L ratio in *Cornus wilsoniana* oil by focusing on FAD2, the microsomal ω-6 desaturase that directly catalyzes oleate-to-linoleate conversion. The fatty-acid profile showed that C18 unsaturated fatty acids dominated the oil composition, with linoleic and oleic acids constituting the principal components. Such a composition implies a strong flux through the C18:1-to-C18:2 conversion step, thereby supporting FAD2 as a logical and mechanistically proximal target for explaining and potentially manipulating the O/L ratio in *C. wilsoniana*.

A key finding is that sequence divergence among the four annotated FAD2 homologs is not diffuse across the entire protein but rather concentrates within a short segment around residues 160–185. As shown in Figure S1, this segment lies in close proximity to the tunnel mouth and pocket boundary, providing a structural rationale for how local sequence changes in this region can propagate to differences in access-pathway behavior observed during simulations. This region exhibited limited deletion in CW09G04700 and an extensive deletion in CW04G07690, and these differences translated into clear structural consequences in the corresponding homology models. The observation that CW04G07690 loses secondary-structure elements in the divergent segment suggests that this region contributes to maintaining the integrity of the catalytic architecture. Importantly, Ramachandran validation indicated that the models are of acceptable stereochemical quality, supporting their use as comparative templates for subsequent docking and dynamics analyses while acknowledging that membrane proteins remain challenging targets for high-accuracy modeling [29,30].

Docking results further suggest that the divergent segment can propagate to functional readouts at the level of substrate accommodation. The markedly weaker binding predicted for CW04G07690 was accompanied by a visibly reduced catalytic pocket, consistent with the hypothesis that large deletions disrupt the pocket environment required for stable oleate positioning. Excluding CW04G07690 from downstream simulations was therefore a pragmatic step to avoid over-interpreting a complex that already showed weak docking performance and lacked stabilizing hydrogen bonds. Among the remaining three homologs, CW02G01750 exhibited the strongest docking affinity, whereas CW09G04700 ranked lowest, a pattern consistent with their subsequent dynamic behaviors.

Molecular dynamics simulations provided a dynamic complement to static docking scores by capturing stability, compactness, residue flexibility, and access-pathway behavior over time [31,32]. CW02G01750 and CW09G27260 converged to relatively stable RMSD regimes and displayed lower, more uniform residue fluctuations across the protein core, with mobility largely confined to terminal regions. In contrast, CW09G04700 showed elevated RMSF within an internal region around residues 180–220 and sustained mild fluctuations in global metrics, which is consistent with the replacement of an α-helical element by a more flexible coil-like segment in the divergent region. Notably, increased local flexibility within the protein core is more likely to affect functional architecture than terminal motions, as it may perturb the geometry and stability of the catalytic pocket and its surrounding access pathway [33]. Consistently, tunnel analysis with quantitative descriptors showed that CW02G01750 and CW09G27260 tended to maintain higher throughput scores and wider bottleneck radii across the sampled snapshots, whereas CW09G04700 displayed greater variability in bottleneck size and access-route geometry, accompanied by repeated ligand readjustments. These results support the view that catalytic competence depends not only on initial binding but also on maintaining an accommodating access pathway that stabilizes substrate positioning during the catalytic cycle.

Importantly, the functional contribution of a given FAD2 homolog to the mature-oil O/L ratio in vivo is determined not only by structural competence inferred from simulations but also by expression level, developmental timing, ER localization, and regulatory context. Therefore, the present results should be interpreted as a structure- and dynamics-based prioritization of candidate FAD2 enzymes, rather than definitive evidence of physiological dominance.

A key limitation is that FAD2 is an endoplasmic-reticulum membrane protein, whereas our simulations were conducted in aqueous solvent without a lipid bilayer. In a native membrane context, lipid-mediated constraints may reshape the tunnel entrance and modulate transient opening events; thus, the tunnel geometries reported here should be viewed as comparative indicators under a simplified environment rather than quantitatively physiological descriptors. Another limitation is that the MD-based inferences were derived from single 80 ns trajectories for each complex; accordingly, the MD outcomes were interpreted as comparative, trend-level evidence supported by concordant patterns across multiple observables. Future work should include membrane-embedded simulations, replicate trajectories, and activity assays to test the proposed access-pathway mechanism and the predicted functional hierarchy.

From an applied perspective, the combined evidence supports CW02G01750 as a strong candidate for a major functional FAD2 in *C. wilsoniana* and suggests that CW09G27260 may serve as a secondary contributor with comparable structural robustness. CW09G04700 appears capable of binding oleic acid but may do so with reduced stability due to increased internal flexibility and a less persistent access pathway. CW04G07690, given its extensive deletion and weak docking performance, may represent a low-activity or conditionally active homolog, or could be a pseudogene-like variant, although functional validation would be required to distinguish these possibilities. Future work integrating expression profiling during oil accumulation, membrane-mimetic simulations, and enzymatic assays will be important to test the predicted functional hierarchy and refine the mechanistic link between access-pathway dynamics and oleate desaturation efficiency.

## 5. Conclusions

In this study, we profiled the fatty-acid composition of *Cornus wilsoniana* oil and compared four annotated FAD2 homologs to identify structural features potentially governing the oleic-to-linoleic conversion step relevant to the O/L ratio. GC-FID confirmed that C18 unsaturated fatty acids dominated the oil, supporting FAD2 as a proximal mechanistic target. A short variable segment accounted for most sequence divergence and produced distinct local structural effects. Docking indicated that CW04G07690 carries a compromised catalytic pocket and weak oleic-acid binding, and it was therefore excluded from downstream analyses. For the remaining homologs, 80 ns molecular dynamics simulations coupled with tunnel analysis revealed that CW02G01750 and CW09G27260 maintain greater conformational stability and increasingly accessible catalytic architectures, whereas CW09G04700 shows elevated internal flexibility and less persistent access pathways with repeated ligand conformational adjustments. Collectively, these findings rank CW02G01750 as a leading structural and dynamic candidate among the annotated FAD2 homologs in C. wilsoniana, with CW09G27260 likely contributing in a complementary manner. Importantly, the present study is limited by the lack of experimental validation at the expression and enzyme-activity levels; therefore, our conclusions represent a structure- and dynamics-based prioritization rather than definitive evidence of physiological dominance or in vivo flux control. Future work integrating expression profiling during oil accumulation and heterologous activity assays, ideally in membrane-mimetic contexts, will be necessary to validate the predicted functional hierarchy and establish a direct link between access-pathway dynamics and oleate desaturation efficiency. This work provides a structure-informed foundation for future efforts aimed at validating and improving the O/L ratio and oil quality in woody oil crops.

## Data Availability

The original contributions presented in this study are included in the article and the Supplementary Materials.

## Supplementary Materials

The following supporting information can be downloaded at: https://www.mdpi.com/article/10.3390/biology15020145/s1, Figure S1: Spatial relationship between the divergent segment and the dominant access tunnel at the end of molecular dynamics simulations.

## References

[1] Chen Y.Z., Gao Z.Z., Yang Y., Liu Q., Jiang L.J., Chen J.Z., Zhou X., Zhang L.H., Ji Y.N., Tu J. (2025). Revealing the Existence of Diverse Strategies for Phosphorus Solubilization and Acquisition in Plant-Growth Promoting Streptomyces misionensis SwB1. Microorganisms.

[2] Liu R.T., Yi J., Li S.H., Xia J.L., Li M., Ding H.Y., Xu L.N., Yang X.H., Yao N. (2021). Design and Preparation Technology of Single/Dual-Cross-Linking Copolymers Based on Swida wilsoniana Oil. ACS Omega.

[3] Halimi R.A., Raymond C.A., Barkla B.J., Mayes S., King G.J. (2022). Development of Selection Indices for Improvement of Seed Yield and Lipid Composition in Bambara Groundnut (*Vigna subterranea* (L.) Verdc.). Foods.

[4] Sabbahi R., Azzaoui K., Rhazi L., Ayerdi-Gotor A., Aussenac T., Depeint F., Taleb M., Hammouti B. (2023). Factors Affecting the Quality of Canola Grains and Their Implications for Grain-Based Foods. Foods.

[5] Rashid M.T., Liu K.L., Han S.M., Jatoi M.A. (2022). The Effects of Thermal Treatment on Lipid Oxidation, Protein Changes, and Storage Stabilization of Rice Bran. Foods.

[6] Shen Y., Shen Y., Liu Y.H., Bai Y., Liang M., Zhang X.Y., Chen Z.D. (2023). Characterization and functional analysis of AhGPAT9 gene involved in lipid synthesis in peanut (*Arachis hypogaea* L.). Front. Plant Sci..

[7] Qin J.S., Kurt E., Lbassi T., Sa L., Xie D.M. (2023). Biotechnological production of omega-3 fatty acids: Current status and future perspectives. Front. Microbiol..

[8] Hajiahmadi Z., Abedi A., Wei H., Sun W.B., Ruan H.H., Qiang Z.G., Movahedi A. (2020). Identification, evolution, expression, and docking studies of fatty acid desaturase genes in wheat (*Triticum aestivum* L.). BMC Genom..

[9] Smith B.E., Lu C.F. (2024). Heat stress during reproductive stages reduces camelina seed productivity and changes seed composition. Heliyon.

[10] Asadi A., Shariati V., Mousavi S., Mariotti R., Mazinani M.H. (2023). Meta-analysis of transcriptome reveals key genes relating to oil quality in olive. BMC Genom..

[11] He Z.X., Chao H.Y., Zhou X.K., Ni Q.Y., Hu Y.M., Yu R.R., Wang M.H., Li C.Z., Chen J.Z., Chen Y.Z. (2023). A chromosome-level genome assembly provides insights into *Cornus wilsoniana* evolution, oil biosynthesis, and floral bud development. Hortic. Res..

[12] Sun D.Q., Quan W.Z., Wang D., Cui J.Y., Wang T.Y., Lin M., Wang Y.J., Wang N., Dong Y.Y., Li X.W. (2022). Genome-Wide Identification and Expression Analysis of Fatty Acid Desaturase (FAD) Genes in *Camelina sativa* (L.) Crantz. Int. J. Mol. Sci..

[13] Nan S.Z., Zhang L.J., Hu X.W., Miao X.M., Han X.X., Fu H. (2021). Transcriptomic Analysis Reveals Key Genes Involved in Oil and Linoleic Acid Biosynthesis during *Artemisia sphaerocephala* Seed Development. Int. J. Mol. Sci..

[14] Shi P., Hua W., Htwe Y.M., Zhang D.P., Li J., Wang Y. (2021). Abscisic Acid Improves Linoleic Acid Accumulation Possibly by Promoting Expression of *EgFAD2* and Other Fatty Acid Biosynthesis Genes in Oil Palm Mesocarp. Front. Plant Sci..

[15] Eberhardt J., Santos-Martins D., Tillack A.F., Forli S. (2021). AutoDock Vina 1.2.0: New Docking Methods, Expanded Force Field, and Python Bindings. J. Chem. Inf. Model..

[16] Laskowski R.A., Swindells M.B. (2011). LigPlot+: Multiple Ligand-Protein Interaction Diagrams for Drug Discovery. J. Chem. Inf. Model..

[17] Van der Spoel D., Lindahl E., Hess B., Groenhof G., Mark A.E., Berendsen H.J.C. (2005). GROMACS: Fast, flexible, and free. J. Comput. Chem..

[18] Yang L., Tian X.L., Gui L.Y., Wang F.L., Zhang G.H. (2023). Key Amino Acid Residues Involved in Binding Interactions between Bactrocera minax Odorant-Binding Protein 3 (BminOBP3) and Undecanol. Insects.

[19] Ji B.H., He X.B., Zhang Y.Z., Zhai J.C., Man V.H., Liu S.H., Wang J.M. (2021). Incorporating structural similarity into a scoring function to enhance the prediction of binding affinities. J. Cheminform..

[20] Ramirez-Silva L., Hernandez-Alcantara G., Guerrero-Mendiola C., Gonzalez-Andrade M., Rodriguez-Romero A., Rodriguez-Hernandez A., Lugo-Munguia A., Gomez-Coronado P.A., Rodriguez-Mendez C., Vega-Segura A. (2022). The K^+^-Dependent and -Independent Pyruvate Kinases Acquire the Active Conformation by Different Mechanisms. Int. J. Mol. Sci..

[21] Diagouraga B., Tambones I., Carivenc C., Bechara C., Nadal M., de Massy B., le Maire A., Robert T. (2024). The TOPOVIBL meiotic DSB formation protein: New insights from its biochemical and structural characterization. Nucleic Acids Res..

[22] Shen L., Liu F.X., Huang L., Liu G.Y., Zhou L.Q., Peng L.H. (2022). VDA-RWLRLS: An anti-SARS-CoV-2 drug prioritizing framework combining an unbalanced bi-random walk and Laplacian regularized least squares. Comput. Biol. Med..

[23] Bahsi M., Di Simone S.C., Zheleva-Dimitrova D., Zengin G., Cusumano G., Flores G.A., Angelini P., Emiliani C., Cetiz M.V., Chiavaroli A. (2025). Unlocking New Pharma/Nutraceutical Frontiers with Neuroprotective Properties of Three *Hypericum* Species: A Study Combination with In Vitro and In Silico Methodologies. Food Sci. Nutr..

[24] Choudhury A., Chandra A., Dawoud T.M., Nafidi H.A., Singh N., Bourhia M. (2023). Immunoinformatics and reverse vaccinology approach in designing a novel highly immunogenic multivalent peptide-based vaccine against the human monkeypox virus. Front. Mol. Biosci..

[25] Autiero I., Vitagliano L. (2023). Enhanced molecular dynamic simulation studies unravel long-range effects caused by sequence variations and partner binding in RNA aptamers. Mol. Ther.-Nucleic Acids.

[26] Oyebamiji A.K., Akintelu S.A., Afolabi S.O., Akintayo E.T., Akintayo C.O., Ebenezer O. (2025). Computer aided study on cyclic tetrapeptide based ligands as potential inhibitors of Proplasmepsin IV. Sci. Rep..

[27] Kamal M.M., Teeya S.T., Rahman M.M., Talukder M.E.K., Sarmin S., Wani T.A., Hasan M.M. (2024). Prediction and assessment of deleterious and disease causing nonsynonymous single nucleotide polymorphisms (nsSNPs) in human FOXP4 gene: An in-silico study. Heliyon.

[28] Zhang K.H., Wu H., Hoppe N., Manglik A., Cheng Y.F. (2022). Fusion protein strategies for cryo-EM study of G protein-coupled receptors. Nat. Commun..

[29] Panigrahi R., Glover J.N.M., Nallusamy S. (2023). A look into DGAT1 through the EM lenses. Biochim. Biophys. Acta-Biomembr..

[30] Ledwitch K., Künze G., Okwei E., Sala D., Meiler J. (2024). Non-canonical amino acids for site-directed spin labeling of membrane proteins. Curr. Opin. Struct. Biol..

[31] Alawi M.M., Gattan H.S., Alharbi A.S., Alruhaili M.H., Al-Zahrani I.A., Masaud S.B.A., Dwivedi V.D., Azhar E.I. (2025). Suppressing lipid biosynthesis in Mycobacterium tuberculosis through polyketide synthase 13 thioesterase inhibition: Insights from computational analysis. J. Infect. Public Health.

[32] Boudou F., Belakredar A., Keziz A., Aissani L., Alsaeedi H., Cronu D., Bechelany M., Barhoum A. (2025). Therapeutic potential of *Curcuma longa* against monkeypox: Antioxidant, anti-inflammatory, and computational insights. Front. Chem..

[33] Smith C.J., Eavis H., Briggs C., Henrici R., Karpiyevich M., Ansbro M.R., Hoshizaki J., van Noort G.J.V., Ascher D.B., Sutherland C.J. (2025). Drug resistance-associated mutations in Plasmodium UBP-1 disrupt its essential deubiquitinating activity. J. Biol. Chem..

