# Supplementary figures and images for: Catalytic Tunnel Dynamics Explain Functional Divergence of FAD2 Homologs Underlying the Oleic-to-Linoleic Ratio in Cornus wilsoniana
Source: Biology (Basel). 2026 Jan 14;15(2):145. doi: 10.3390/biology15020145 (PMC12838259; doi:10.3390/biology15020145)

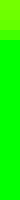

Supplement: Supplementary file 1 [file biology-15-00145-s001.zip › caver_output/CW02G01750/0 ns/analysis/profile_heat_maps/average_images/cl_000001_average_heat_map.png]

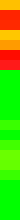

Supplement: Supplementary file 1 [file biology-15-00145-s001.zip › caver_output/CW02G01750/0 ns/analysis/profile_heat_maps/average_images/cl_000002_average_heat_map.png]

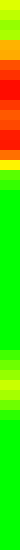

Supplement: Supplementary file 1 [file biology-15-00145-s001.zip › caver_output/CW02G01750/0 ns/analysis/profile_heat_maps/average_images/cl_000003_average_heat_map.png]

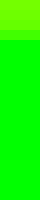

Supplement: Supplementary file 1 [file biology-15-00145-s001.zip › caver_output/CW02G01750/0 ns/analysis/profile_heat_maps/cl_000001_profile_heat_map.png]

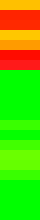

Supplement: Supplementary file 1 [file biology-15-00145-s001.zip › caver_output/CW02G01750/0 ns/analysis/profile_heat_maps/cl_000002_profile_heat_map.png]

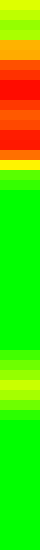

Supplement: Supplementary file 1 [file biology-15-00145-s001.zip › caver_output/CW02G01750/0 ns/analysis/profile_heat_maps/cl_000003_profile_heat_map.png]

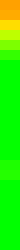

Supplement: Supplementary file 1 [file biology-15-00145-s001.zip › caver_output/CW02G01750/20 ns/analysis/profile_heat_maps/average_images/cl_000001_average_heat_map.png]

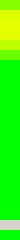

Supplement: Supplementary file 1 [file biology-15-00145-s001.zip › caver_output/CW02G01750/20 ns/analysis/profile_heat_maps/average_images/cl_000002_average_heat_map.png]

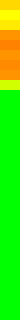

Supplement: Supplementary file 1 [file biology-15-00145-s001.zip › caver_output/CW02G01750/20 ns/analysis/profile_heat_maps/average_images/cl_000003_average_heat_map.png]

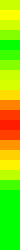

Supplement: Supplementary file 1 [file biology-15-00145-s001.zip › caver_output/CW02G01750/20 ns/analysis/profile_heat_maps/average_images/cl_000004_average_heat_map.png]

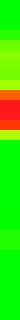

Supplement: Supplementary file 1 [file biology-15-00145-s001.zip › caver_output/CW02G01750/20 ns/analysis/profile_heat_maps/average_images/cl_000005_average_heat_map.png]

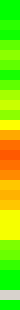

Supplement: Supplementary file 1 [file biology-15-00145-s001.zip › caver_output/CW02G01750/20 ns/analysis/profile_heat_maps/average_images/cl_000006_average_heat_map.png]

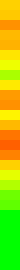

Supplement: Supplementary file 1 [file biology-15-00145-s001.zip › caver_output/CW02G01750/20 ns/analysis/profile_heat_maps/average_images/cl_000007_average_heat_map.png]

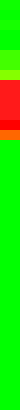

Supplement: Supplementary file 1 [file biology-15-00145-s001.zip › caver_output/CW02G01750/20 ns/analysis/profile_heat_maps/average_images/cl_000008_average_heat_map.png]

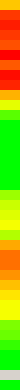

Supplement: Supplementary file 1 [file biology-15-00145-s001.zip › caver_output/CW02G01750/20 ns/analysis/profile_heat_maps/average_images/cl_000009_average_heat_map.png]

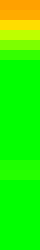

Supplement: Supplementary file 1 [file biology-15-00145-s001.zip › caver_output/CW02G01750/20 ns/analysis/profile_heat_maps/cl_000001_profile_heat_map.png]

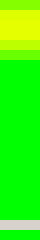

Supplement: Supplementary file 1 [file biology-15-00145-s001.zip › caver_output/CW02G01750/20 ns/analysis/profile_heat_maps/cl_000002_profile_heat_map.png]

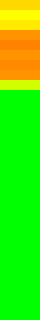

Supplement: Supplementary file 1 [file biology-15-00145-s001.zip › caver_output/CW02G01750/20 ns/analysis/profile_heat_maps/cl_000003_profile_heat_map.png]

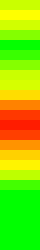

Supplement: Supplementary file 1 [file biology-15-00145-s001.zip › caver_output/CW02G01750/20 ns/analysis/profile_heat_maps/cl_000004_profile_heat_map.png]

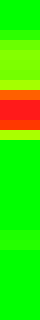

Supplement: Supplementary file 1 [file biology-15-00145-s001.zip › caver_output/CW02G01750/20 ns/analysis/profile_heat_maps/cl_000005_profile_heat_map.png]

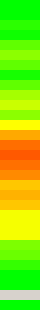

Supplement: Supplementary file 1 [file biology-15-00145-s001.zip › caver_output/CW02G01750/20 ns/analysis/profile_heat_maps/cl_000006_profile_heat_map.png]

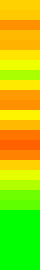

Supplement: Supplementary file 1 [file biology-15-00145-s001.zip › caver_output/CW02G01750/20 ns/analysis/profile_heat_maps/cl_000007_profile_heat_map.png]

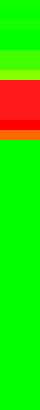

Supplement: Supplementary file 1 [file biology-15-00145-s001.zip › caver_output/CW02G01750/20 ns/analysis/profile_heat_maps/cl_000008_profile_heat_map.png]

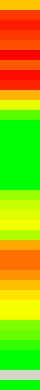

Supplement: Supplementary file 1 [file biology-15-00145-s001.zip › caver_output/CW02G01750/20 ns/analysis/profile_heat_maps/cl_000009_profile_heat_map.png]

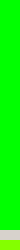

Supplement: Supplementary file 1 [file biology-15-00145-s001.zip › caver_output/CW02G01750/40 ns/analysis/profile_heat_maps/average_images/cl_000001_average_heat_map.png]

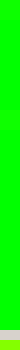

Supplement: Supplementary file 1 [file biology-15-00145-s001.zip › caver_output/CW02G01750/40 ns/analysis/profile_heat_maps/average_images/cl_000002_average_heat_map.png]

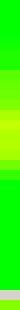

Supplement: Supplementary file 1 [file biology-15-00145-s001.zip › caver_output/CW02G01750/40 ns/analysis/profile_heat_maps/average_images/cl_000003_average_heat_map.png]

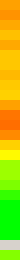

Supplement: Supplementary file 1 [file biology-15-00145-s001.zip › caver_output/CW02G01750/40 ns/analysis/profile_heat_maps/average_images/cl_000004_average_heat_map.png]

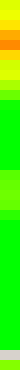

Supplement: Supplementary file 1 [file biology-15-00145-s001.zip › caver_output/CW02G01750/40 ns/analysis/profile_heat_maps/average_images/cl_000005_average_heat_map.png]

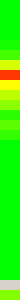

Supplement: Supplementary file 1 [file biology-15-00145-s001.zip › caver_output/CW02G01750/40 ns/analysis/profile_heat_maps/average_images/cl_000006_average_heat_map.png]

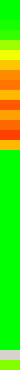

Supplement: Supplementary file 1 [file biology-15-00145-s001.zip › caver_output/CW02G01750/40 ns/analysis/profile_heat_maps/average_images/cl_000007_average_heat_map.png]

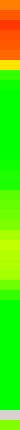

Supplement: Supplementary file 1 [file biology-15-00145-s001.zip › caver_output/CW02G01750/40 ns/analysis/profile_heat_maps/average_images/cl_000008_average_heat_map.png]

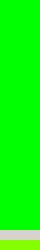

Supplement: Supplementary file 1 [file biology-15-00145-s001.zip › caver_output/CW02G01750/40 ns/analysis/profile_heat_maps/cl_000001_profile_heat_map.png]

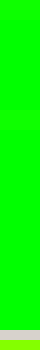

Supplement: Supplementary file 1 [file biology-15-00145-s001.zip › caver_output/CW02G01750/40 ns/analysis/profile_heat_maps/cl_000002_profile_heat_map.png]

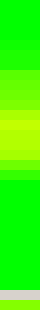

Supplement: Supplementary file 1 [file biology-15-00145-s001.zip › caver_output/CW02G01750/40 ns/analysis/profile_heat_maps/cl_000003_profile_heat_map.png]

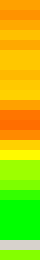

Supplement: Supplementary file 1 [file biology-15-00145-s001.zip › caver_output/CW02G01750/40 ns/analysis/profile_heat_maps/cl_000004_profile_heat_map.png]

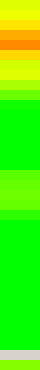

Supplement: Supplementary file 1 [file biology-15-00145-s001.zip › caver_output/CW02G01750/40 ns/analysis/profile_heat_maps/cl_000005_profile_heat_map.png]

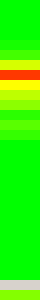

Supplement: Supplementary file 1 [file biology-15-00145-s001.zip › caver_output/CW02G01750/40 ns/analysis/profile_heat_maps/cl_000006_profile_heat_map.png]

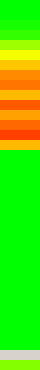

Supplement: Supplementary file 1 [file biology-15-00145-s001.zip › caver_output/CW02G01750/40 ns/analysis/profile_heat_maps/cl_000007_profile_heat_map.png]

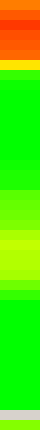

Supplement: Supplementary file 1 [file biology-15-00145-s001.zip › caver_output/CW02G01750/40 ns/analysis/profile_heat_maps/cl_000008_profile_heat_map.png]
