# Supplementary material for: Catalytic Tunnel Dynamics Explain Functional Divergence of FAD2 Homologs Underlying the Oleic-to-Linoleic Ratio in Cornus wilsoniana
Source: Biology (Basel). 2026 Jan 14;15(2):145. doi: 10.3390/biology15020145 (PMC12838259; doi:10.3390/biology15020145)
Supplement: Supplementary file 1 [file biology-15-00145-s001.zip › Supplementary document.pdf]

Supporting Information

Catalytic tunnel dynamics explain functional divergence of FAD2 homologs underlying the oleic-to-linoleic ratio in *Cornus wilsoniana*

Wenyan Yuan[a,b,1], Wei Wu[a,1], Shushan Jia[c], Yunzhu Chen[a], Peiwang Li[a], Yan Yang[a], Lijuan Jiang[b], Qiang Liu[b], Li Li[a], Wenbin Zeng[b], Xiao Zhou[a,*], Jingzhen Chen[a,*]

[a] State Key Laboratory of Woody Oil Resources Utilization, Hunan Academy of Forestry, Changsha, Hunan 410004, PR China

[b] College of Life Science and Technology, Central South University of Forestry and Technology, 498 South Shaoshan Road, Changsha, Hunan 410004, PR China

[c] Qingdao Research Academy of Environmental Sciences, Qingdao, Shandong 266000, PR China

>CW02G01750

MGAGGRMSAPPASKKPESANLRRVPYSKPPFTLGEIKKAIPPHCFQRSVLHSFS
YVIYDLTIAFIFYYIATNYIHLIPSPLSFLAWPVYWIFQGCILTGVWVIAHECGH
HAFSDYQSLDDTVGLILHSALLVPYFSWKYSHRRHHSNTASLERDEVFVPKH
KFEMKWYAKYLNNPLGRVFTLVVQLTLGWPLYLLCNVSGRHYNRFACHFDP
YGPIYSDRERLQIYLSDAGVLAVTYGLYRVAAAKGLAWVLCVYGVPLLIVNG
FLVLITYLQHTHPALPHYDSSEWDWLRGALATVDRDYGILNKVFHHITDTHVA
HHLISTMPHYHAMEATKAIKPILGDYYQFDGTPVFKAMWREAKECIYVEADE
GNQSTGVFWYNNKL

>CW09G27260

MGAGGQMSVPPESKQQESDVLQRVPCSKPPFTLGEIKKAIPSNCFNRSLVHSFS

YVVFDLTIAFIFYYIATNYIHLLPYPLSLVAWPVYWIFQGCVLTGVWVIAHECG

HHAFSDYQWLDDTVGLILHSALLVPYFSWKYSHRRHHSNTGSLDRDEVFVPR

LKSRITWFAKYLNNPPGRVFTLVLQLTLGWPLYLLFNVSGRQYDRFACHYDPY

GPIYSDRERIQIYLSDAGVLAVTCGLYRLMAAKGLAWVVCVYGGPLLIVNGFL

VLITYLQHTHPSLPHYDSSEWDWLRGALATVDRDYGILNRVFHNITDTHVAH

HLFSTMPHYHAMEATKAIKPILGDYYQFDGTPIIKAMWREAKECIYVEADKG

DQNKGVFWYRKKL

>CW09G04700

MGAGGRMSVTKTESEEEHKNPIPRVPHSKPPFTLGEIKKAIPPHCFKRSLLRSF

SYVVYDLTTALLLYYIATSYFHLLPQPFRALAWPLYWALQGCILTGVWVIAHE

CGHHAFSDYQWVDDTVGLILHSFLLVPYFSWKYSHRRHHSNTGSIEHDEVFV

PKLKSKMDGRIVTLVVTLTLGWPLYLAFNVSGRPYDRFACHFHPNSPIYNDRE

RLQIYISDAGIMAATYVLYRLVLAKGLAWVVCVYGVPLLIVNSFLVLITYLQH

THPALPHYDSSEWDWLRGALATVDRDYGVLNKVFHNITDTHVAHHLFSTMP

HYNAMEATKAIKPILGEYYRFDGTPFYKAMWREARECLYVESDDGTPSNGVL

WYKNKF

>CW04G07690

MSESKKPGSANFGRVPYSKPPFTLGEIKKAIPPHCFQRFVLHSFYYVIHDLTIAF

IFYYIATNYIHLLPYPLSFVAWPVYWIFQGCVLSGVWVIGHECGHHAFSDYQW

LDDTVGFILHTALLVPYFSWKYSHRRHHSNIGSLKGDDAHVAKHKLELRCQR

LQISLSDVGVLGVTYGLYRLAAAKGLAWVLCVYGVPLLIVNGFFVLLTYLHH

THPSLPRYDSPEWDWLRGSLATVDRDYGILNKVFHHITDTHVTHHLFSTMPH

YHATEATKAIKPILRDYYLFDGTPFFKAFWREAKECIYVEADEGNQSKGVFW

YNNNL

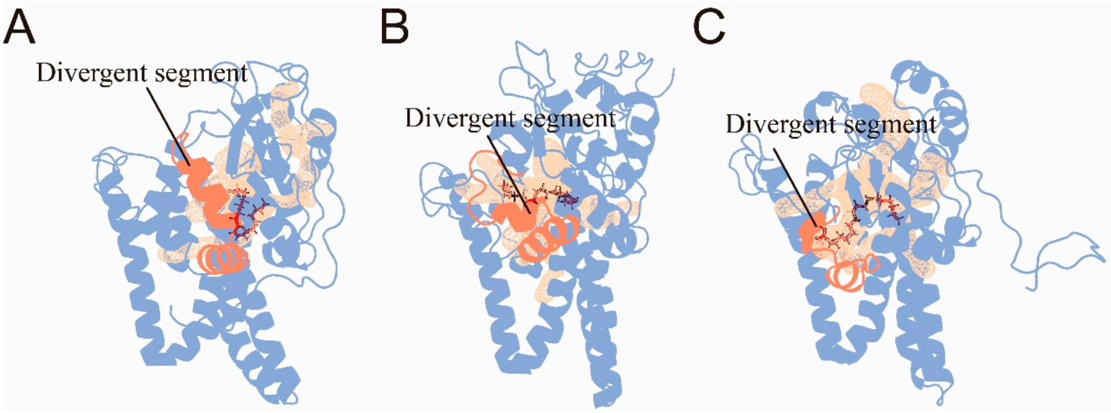

Figure. S1. Spatial relationship between the divergent segment and the dominant access tunnel at the end of molecular dynamics simulations. Panels A–C show the 80 ns end-point structures of the oleic-acid complexes for CW02G01750, CW09G27260, and CW09G04700, respectively. The divergent segment spanning residues 160–185 is highlighted in red, and the dominant access tunnel predicted by CAVER is shown in yellow. Oleic acid is displayed as sticks.
